# Engineered Protein Polymer-Gold Nanoparticle Hybrid Materials for Small Molecule Delivery

**DOI:** 10.4172/2157-7439.1000356

**Published:** 2016-02-29

**Authors:** Min Dai, JA Frezzo, E Sharma, R Chen, N Singh, C Yuvienco, E Caglar, S Xiao, A Saxena, JK Montclare

**Affiliations:** 1 Department of Chemical and Biomolecular Engineering, NYU Tandon School of Engineering, Brooklyn, New York 11201, USA; 2 Department of Biology, Brooklyn College and Graduate Center, City University of New York, Brooklyn, New York 11210, USA; 3 Department of Chemistry, New York University, New York, New York 10003, USA; 4 Department of Biochemistry, SUNY Downstate Medical Center, Brooklyn, New York 11203, USA

**Keywords:** Proteins, Gold nanoparticles, Drug delivery, Nanocomposites

## Abstract

We have fabricated protein polymer-gold nanoparticle (P-GNP) nanocomposites that exhibit enhanced binding and delivery properties of the small hydrophobic molecule drug, curcumin, to the model breast cancer cell line, MCF-7. These hybrid biomaterials are constructed via *in situ* GNP templated-synthesis with genetically engineered histidine tags. The P-GNP nanocomposites exhibit enhanced small molecule loading, sustained release and increased uptake by MCF-7 cells. When compared to the proteins polymers alone, the P-GNPs demonstrate a greater than 7-fold increase in curcumin binding, a nearly 50% slower release profile and more than 2-fold increase in cellular uptake of curcumin. These results suggest that P-GNP nanocomposites serve as promising candidates for drug delivery vehicles.

## Introduction

The fabrication of multifunctional, stimuli-responsive organic-inorganic hybrid materials that can self-assemble into defined structures bears tremendous potential in drug delivery and medicine [1–4]. The synthesis of hybrids combining stimuli responsive materials and gold nanoparticles (GNPs) has been explored in large part due to their unique properties [5–9]. For example, a composite hydrogel material comprised of temperature-sensitive copolymers, N-isopropylacrylamide and acrylamide, embedded with GNPs, bearing a gold sulfide nanoshell designed to absorb and convert near-IR light to heat has been developed [10]. Upon light triggered activation, the copolymer, when entrapped with a small molecule drug, undergoes a conformational change that in turn leads to drug release [10]. Another example of using gold nanoparticles for triggered drug release relies on liposomal nanoparticles composed 1,2-dipalmitoylsn-glycero-3-phosphocholine, 1-palmitoyl-2-hydroxy-sn-glycero-3-phosphocholine and 1,2-distearoyl-sn-glycero-3-phosphoethanolamine-N-[carboxy(polyethylene glycol)-2000] [11]. Such liposomes, when loaded with both GNPs and the hydrophilic drug calcein, when subjected to 532 nm laser treatments leads to light controlled calcein release due to microbubble cavitation of the liposome membrane[11]. While a wide range of synthetic materials have been developed and explored [10,12–14], proteins have attracted intense attention due to the fine molecular and conformational control of sequence and structure [15,16]. Recently, GNPs have been decorated with a library of cationic groups that complex non-covalently with green fluorescent protein (GFP) [17]. These GFP-GNP complexes have been employed in an array to chemically detect human serum proteins in complex serum. In this case, the strategy for construction of such protein-GNP hybrids rely on the covalent or non-covalent linkage of chemically pre-fabricated GNPs that have been synthesized under harsh organic solvents [18,19].

**Figure 1:**
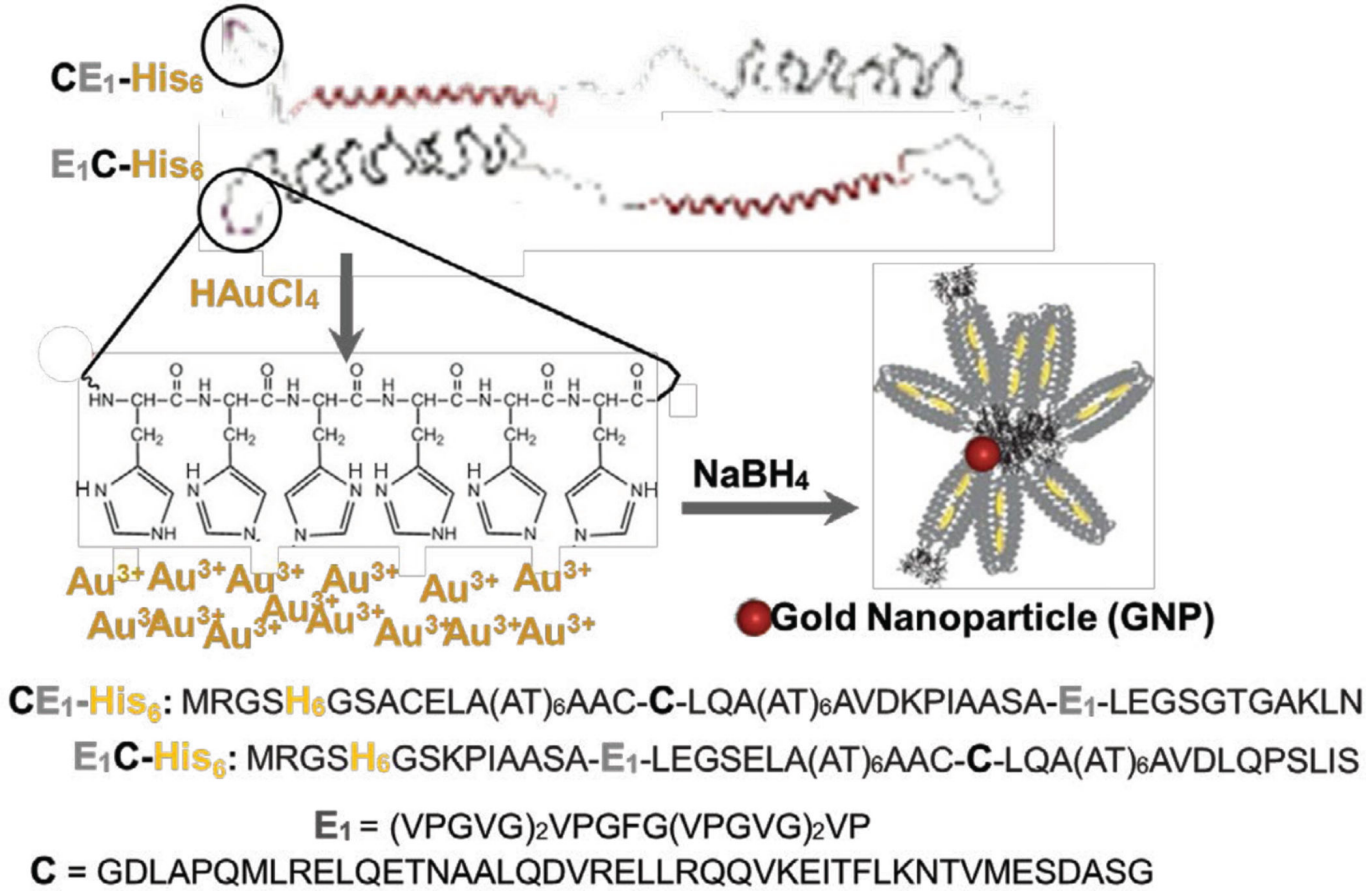
Protein polymer sequences of CE_1_-His_6_ and E_1_C -His_6_ and gold nanoparticle (GNP) templated-synthesis strategy.

Specific chemical transformations are employed to prepare GNPs of discrete sizes and additional chemical steps are required to further decorate GNPs with key ligands as well as conjugate them with the macromolecule of interest [20]. Yet biological systems are able to fabricate GNPs under ambient conditions *in situ* through specific sequences [19,21–26]. We seek to generate multifunctional *protein materials* capable of: (i) templated-synthesis of inorganic nanoparticles *in situ* to fabricate organic-inorganic hybrids without the need for covalent bonding between each substituent part; (ii) encapsulating and stabilizing large payloads of small molecules; and (iii) modulating the delivery of small molecule chemotherapeutic drugs in clinically relevant cells (Figure 1).

Previously we have produced protein diblock copolymers comprised of two different self-assembling domains (SADs): 1) an elastin-like peptide (E); and 2) the coiled-coil region of Cartilage Oligomeric Matrix protein (C) [27,28]. While the diblocks, EC and CE, exhibit different temperature dependent conformations and self-assembly [27], they bind to curcumin [28], a naturally occurring anti-inflammatory agent bearing chemoprevention effects [29]. Curcumin has been chosen not only for its chemotherapeutic properties against the breast cancer cell line MCF-7 but also because it is insoluble and degrades rapidly under physiological conditions [29,30]. A drug delivery system that can solubilize and stabilize labile molecules such as curcumin would have beneficial therapeutic applications [29].

Here, we employ the two diblocks E_1_C-His_6_ and CE_1_-His_6_ each bearing an N-terminal hexahistidine tag for the templated-synthesis of gold nanoparticles (GNPs) *in situ* to yield the nanocomposites E_1_C-His_6_-GNP and CE_1_-His_6_-GNP, respectively (Figure 1). These protein polymers are selected due to their thermostability and superior small molecule binding abilities [31]. We hypothesize that such P-GNP nanocomposites will influence the thermoresponsiveness, drug binding capacity and release. Notably, E_1_C-His_6_-GNP and CE_1_-His_6_-GNP demonstrate elevated inverse temperature transitions, improved small molecule loading capacity, sustained release and enhanced uptake by cancer cells when compared to protein polymers alone.

## Materials and Methods

### General

Yeast extract and curcumin were obtained from Acros Organics (Geel, Belgium). Tryptic soy agar and gold(III) chloride trihydrate were acquired from MP Biomedicals (Santa Ana, CA). Ampicillin, isopropyl β-D-1-thiogalactopyranoside (IPTG), imidazole, sodium monobasic phosphate, sodium dibasic phosphate, sodium dodecyl sulfate, sodium hydroxide, sodium chloride, sucrose, tris-hydrochloride, tryptone, PFU high fidelity, DpnI, ACS grade methanol and urea were obtained from Fisher Scientific (Pittsburgh, PA). 4-(2-hydroxyethyl)-1-piperazineethanesulfonic acid (HEPES), magnesium sulfate, nickel chloride, sodium borohydride were purchased from Sigma Aldrich (St. Louis, MO). Tricine was purchased from Alfa Aesar (Ward Hill, MA). Glacial acetic acid and Factor Xa cleavage kit were purchased from EMD Millipore (Rockland, MA). Ethyl acetate was purchased from Pharmco-AAPER (Brookfield, CT). Ethylenediaminetetraacetic acid (EDTA) and hydrochloric acid were acquired from VWR (Radnor, PA). HPLC grade methanol was obtained from Ricca Chemical Company (Arlington, TX). Sephadex™ G-25 medium beads were purchased from Amersham Pharmacia Biotech AB (Piscataway, NJ). Columns were purchased from Bio-Rad (Hercules, CA).

### Site-directed mutagenesis

pQE30/CE_1_ and pQE30/E_1_C were employed for production of CE_1_-His_6_ and E_1_C-His_6_ proteins in this study [31]. In order to generate proteins with Factor Xa IEGR cleavage site, site-directed mutagenesis was performed using the following primers: 5’-cgcagtagcagcgagctcgcgcccttctatgtgatggtgatggt*g*-3’ and 5’-cgcgctagccgcaatgcgcccttctatgtgatggtg atggtg-3’ and their reverse complements to generate PQE30/CE_1_-IGER and PQE30/E_1_C-IGER, respectively. Following the standard protocol for parent strand digestion using Dpn1, the resulting product was transformed into XL1-Blue cells for future use. Mutations were verified by DNA sequencing at Eurofins (Huntsville, AL).

### Protein expression and purification

Biosynthesis and purification of CE_1_-His_6_, E_1_C-His_6_, CE_1_-IEGR and E_1_C-IEGR, was performed as previously described (Figure S1) [31]. Briefly, PQE30/CE_1_, PQE30/E_1_C, PQE30/CE_1_-IGER and PQE30/E_1_C-IGER were used to express the CE_1_-His_6_, E_1_C-His_6_, CE_1_-IEGR and E_1_C-IEGR proteins, respectively. All proteins were purified on a HiTrap IMAC FF column charged with nickel under denaturing conditions. For the negative control, CE_1_-IEGR and E_1_C-IEGR were dialyzed in 10 mM sodium phosphate buffer, pH 8.0, using SnakeSkin dialysis tubing (Thermo Scientific, 3.5 K MWCO). Factor Xa cleaves the protein after IEGR site, removing the His-tag. This reaction occurs in 1 µL of 0.5 unit/µL enzyme, 44 µL protein sample of 0.2 mg/mL concentration and 5 µL cleavage buffer (final cleavage buffer condition is 2 mM Tris-HCl, 50 mM NaCl, 0.5 mM CaCl_2_, pH 7.25). This ratio was scaled up to cleave 4 mL of the samples and cleavage reaction was allowed for 4 days at 4°C. This solution containing cleaved protein, His-tag and Factor Xa was transferred into Factor Xa capture resin and then passed through nickel beads to isolate the cleaved CE_1_ and E_1_C (Figure S2). After confirming the purity using sodium dodecylsulfate-polyacrylamide gel electrophoresis (SDS-PAGE), CE_1_-His_6_, E_1_C-His_6_, CE_1_ and E_1_C were dialyzed into 10 mM sodium phosphate buffer, pH 8.0.

### Gold nanoparticle templated-synthesis

A 0.1 M HAuCl_4_·3H_2_O solution (reactive gold solution) was prepared in dH_2_O. Approximately, 1.2 µL of the reactive gold solution was added into 300 µL of 10 µM protein sample, followed by gentle vortex for 10 minutes at room temperature. To the mixture, a 3.6 µL freshly prepared 0.1 M NaBH_4_ solution in dH_2_O, was added to reduce Au^3+^ to Au^0^. The mixture was then gently rotated to prevent aggregation or uneven templated-synthesis. The reaction was carried at room temperature for 1 hour. The molar ratio of Au^3+^ to protein was 40 to 1, while the NaBH_4_ to Au^3+^ ratio was 2.5 to 1. The resulting protein polymer-gold nanoparticle (P-GNP) nanocomposites were stored at room temperature for 1 hour before further characterization.

### Absorbance spectroscopy

The absorbance spectrum from 200 nm – 1000 nm of each P-GNP nanocomposite was scanned using SpectraMax M2 (Molecular Devices) in UV-transparent 96 well microplate (Corning, half area flat bottom). As a control, buffer, CE_1_-His_6_ and E_1_C-His_6_, in addition to the cleaved CE_1_ and E_1_C proteins at pH 8 were scanned. All protein samples were prepared at 10 µM in 10 mM sodium phosphate buffer, pH 8.0.

### Transmission electron microscopy

Transmission Electron Microscopy (TEM) was used to identify the nanometer-sized structures that resulted from self-assembly at room temperature. Samples were prepared in water at 10 µM concentrations in 10 mM sodium phosphate buffer pH 8.0. The samples were gently mixed and applied on a carbon coated 400 mesh Cu/Rh grids and negatively stained with 1% uranyl acetate as previously described [31]. The images of the samples were collected on a Phillips CM12 TEM instrument at 120 kV. The particle area and size were measured using Image J [32–34]. The protein particle sizes were determined from at least >130 particles, while sizes of the resulting GNPs were determined from at least >130 particles via Image J [32–34]. A histogram of the GNP sizes was generated to determine the average size distribution.

### Circular dichroism (CD) spectroscopy

Wavelength-dependent Circular Dichroism (CD) spectra were collected on a Jasco J-815 CD Spectrometer equipped with a PTC-423S single position Peltier temperature control system and counter-cooled with an Isotemp 3016S (Fisher Scientific) water bath. Samples were loaded in a Hellma 218 quartz cuvette (500 µL, 1 mm path length). A far-UV temperature-dependent wavelength scan from 185–260 nm as a function of temperature was completed for CE_1_-His_6_ and E_1_C-His_6_ in the absence and presence of GNPs at 0.2 mg/mL in 10 mM sodium phosphate buffer pH 8.0 at scan rate of 50 nm/min for a range of temperatures (25–90°C) with 3 accumulation scans. At least two batches of separately purified proteins were measured. CD data was converted into mean residue molar ellipticity (MRW) via equation [θ]_MRW_ = θ·MW/(10·n·C·l), where θ is in mdeg, MW is molecular weight, n is amino acid number in protein, C is concentration in mg/mL, l is path length in cm [35]. Fitting and calculation of protein secondary structure was processed with CDSSTR methods [36–38]. Parameters for the calculation using CDSSTR program were identical to our previously published work [31].

### Turbidometry

The turbidometry, or inverse temperature transition (T_t_), was determined via UV-Vis Spectrophotometer Cary-50 (Agilent Technology) equipped with TC 125 temperature controller (Quantum Northwest) in Type 21 quartz cuvette with 10 mm path length (Buck Science) by monitoring the change in turbidity at 800 nm from 25°C to 80°C at a rate of 1°C/min. Protein stock solutions for T_t_ measurement were prepared in 0.2 mg/mL (or 14.3 µM and 14.4 µM for CE_1_-His_6_ and E_1_C-His_6_, respectively) in 10 mM sodium phosphate buffer, pH 8.0. In order to bring T_t_ value of all the samples into instrument operation range, highly concentrated NaCl solution was added prior to T_t_ measurement (Table S1). Measurements were performed on at least two different protein sample preps to calculate the average T_t_. The T_t_ was determined at the midpoint of the normalized turbidity [39].

### Small molecule loading and release

Curcumin (6.5 nmol final concentration from 3 mM stock solution in HPLC grade methanol) was incubated with 1.3 nmol of CE_1_-His_6_, E_1_C-His_6_, CE_1_-His_6_-GNP and E_1_C-His_6_-GNP at room temperature for 2 hours and loaded onto Bio-Rad Spin6 columns packed with Sephadex G-25 medium beads 0.5 cm high. Bound protein polymer-curcumin complexes (in the presence or absence of GNP) were eluted by size, washed 3 times in 50 µL sodium phosphate buffer, followed by centrifugation for 5 min at 14000 rpm. The beads containing unbound curcumin were collected separately and resuspended back to buffer for solvent extraction. Both bound and unbound curcumin were extracted by adding 150 µL ethyl acetate and quantitatively determined by measuring absorbance at 416 nm. Absorbance was measured in a Hellma 105.201-QS type cuvette (10 mm light path, 100 µL sample) on SpectraMax M2. This binding study was performed on at least three different protein sample preparations to calculate the average loading capacities with errors represented as the standard deviation of the three trials.

Release of curcumin from CE_1_-His_6_, E_1_C-His_6_, CE_1_-His_6_-GNP and E_1_C-His_6_-GNP was then investigated. Curcumin (26 nmol) was added to 200 µL of 26 µM (5.2 nmol) protein sample. After 2 hours of incubation at room temperature, the solution was adjusted to contain a final concentration of 0.5 M NaCl. The protein polymer-curcumin complex (in the presence or absence of GNP) were incubated at 45°C (well above the T_t_) for 30 min and centrifuged to separate protein polymer-curcumin complex from excess curcumin. The pellets were resuspended in 200 µL of 50 mM phosphate buffer, pH 7.4 and kept at room temperature in the dark for release. After 10 min, the suspensions were centrifuged and the supernatant was removed and used for extraction assessment of released curcumin. This resuspension-incubation-spin-release cycle was repeated for the next eight hours at the following time points: 10, 25, 55, 85, 135, 195, 255, 315 and 495 min. Release study was performed on two different protein sample preparations to obtain the averaged release profile. Error bars on the release data represented standard error of the two sample preparations.

### Cell culture studies

MCF7 human breast cancer cells were obtained from ATCC and maintained at 37°C, 5% CO_2_ as monolayer cultures in Dulbecco’s modified Eagle’s medium (DMEM with high glucose containing phenol red) supplemented with 10% (v/v) fetal bovine serum (FBS), gentamicin (50 µg/mL), 100 U penicillin/ 100 µg/mL streptomycin. Because the loading capacities of curcumin for P-GNP nanocomposites are much larger than those of the proteins in the absence of GNP we prepared two corresponding curcumin controls that represented the bound curcumin levels in P-GNP and protein polymers alone (Table S2). To avoid any uptake of unbound curcumin by the cells directly, we limited the curcumin amount that is equivalent to the loading capacity of 26 µM of protein samples in 50 mM sodium phosphate buffer, pH 7.4 and allowed to bind for 2 hours at room temperature prior to cell culture studies.

Multiple sets of experiments were performed to record curcumin uptake by image acquisition using FITC filter (Em: 520 nm) under fluorescence microscopy and direct measurement of curcumin uptake in cell extractions. Cells were grown directly on 24-well culture plates (8 × 10^4^ cells/well) for cell extraction or on cover slips for microscopy. After 24 hours of cell plating, cells were treated for 4 or 24 hours with different combination of proteins with or without GNP and/or curcumin. For all the treatments, the total volume of samples with DMEM in 24-well plates was kept constant at 300 µL, with proteins prepared at 10 µM concentrations. The ratio of sample amount to number of cells was also kept constant. The results are representative of two such independent sets of experiments.

For direct measurement of curcumin uptake, cells were washed with Dulbecco’s phosphate buffered saline and lysed with 200 µL RIPA/ well (25 mM TrisHCl pH7.6, 150 mM NaCl, 1% NP-40, 1% sodium deoxycholate, 0.1% SDS) at room temperature for 20 min with gentle shaking. Lysed cells were then collected and vortexed. For curcumin extraction, 150 µL ethyl acetate was added the lysed cells. Thorough extraction was ensured by violently shaking the lysate-solvent mixture for 30 seconds. Curcumin containing solvent phase was then separated by centrifuging at 14,000 RPM for 2 minutes at room temperature. Absorbance of curcumin in ethyl acetate was measured using SpectraMax M2 (Molecular Devices) in Hellma 105.201-QS type quartz cuvette (100 µL volume, 10 mm light path) at 416 nm [40,41].

For fluorescent imaging of curcumin uptake, cells on coverslips were fixed with 300 µL 4% paraformaldehyde solution in DPBS for 20 minutes at room temperature on a plate rocker [42]. Following fixation, cell-containing coverslips were washed 3 × 300 µL DPBS and were mounted on glass slides using DAPI containing mounting medium (Southern Biotech Dapi Fluoromount-G). Coverslips were then sealed using clear nail polish for viewing under microscopy and long-term storage. Cells were viewed using fluorescence microscope IX71 (Olympus) using DAPI (for cell nuclei) and FITC (for curcumin uptake) at 60× magnification while keeping the exposure time for the FITC images constant at 200 milliseconds.

Cell viability measurements were carried out using a CellTiter 96^®^ Aqueous One solution kit (Promega) in a 96-well plate, seeded 1 × 10^4^ cells/well. After 24 hours, the cells were treated for 4 hours or 24 hours with protein polymers and P-GNP nanocomposites with and without curcumin along with control treatments of curcumin alone and media alone. After the treatment periods, 20 µL [3–(4,5-dimethylthiazol-2-yl)-5-(3-carboxymethoxyphenyl)-2-(4-sulfophenyl)-2H-tetrazolium (MTS) was added to each well, followed by incubation at 37°C for 3 hours. The plate was centrifuged for 3 minutes at 2500 rpm and then subjected to absorbance measurements at 490 nm (Tables S3 and S4).

## Results

### Fabrication of P-GNP nanocomposites

**Figure 2:**
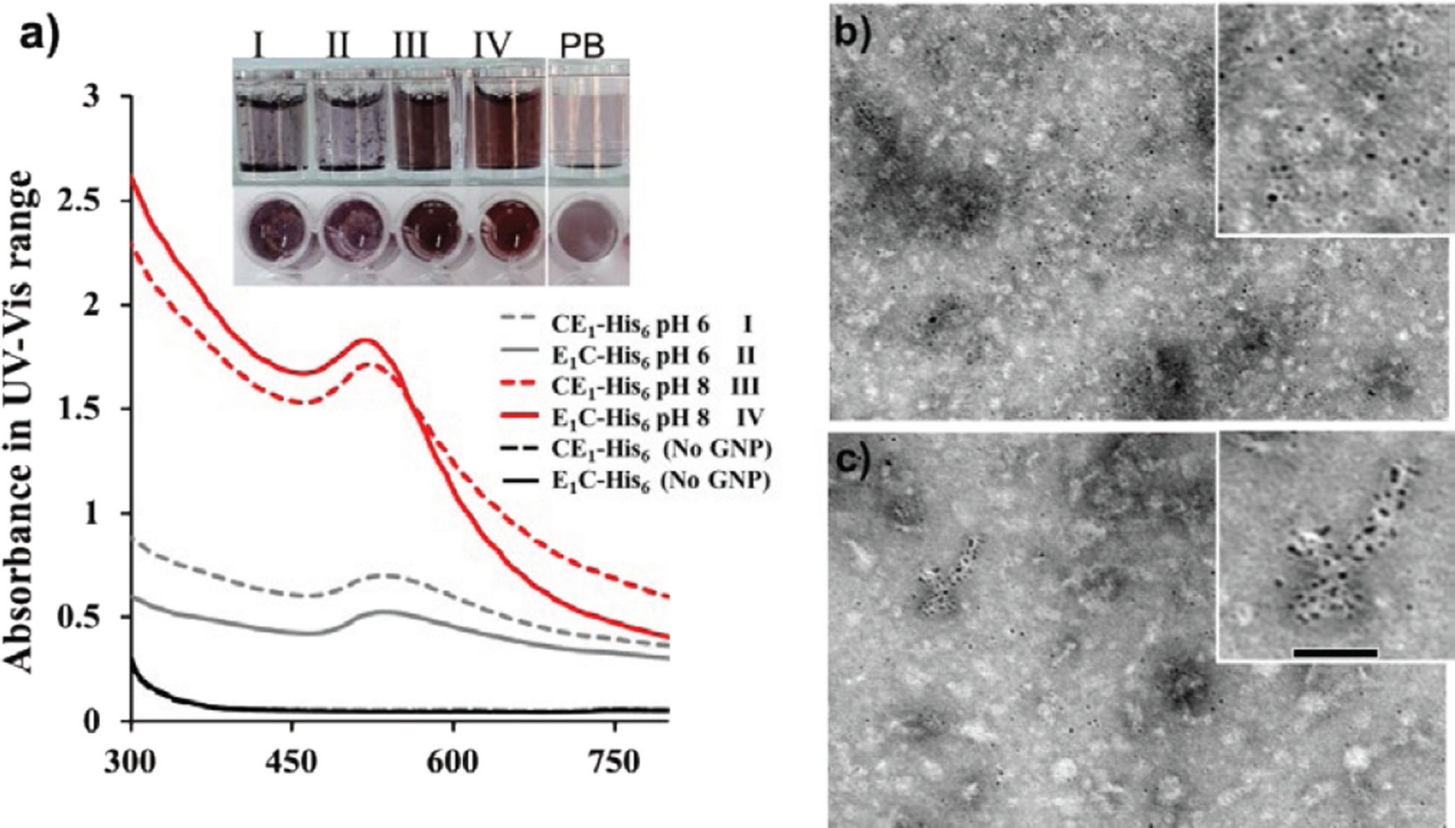
*In situ* gold nanoparticle (GNP) templated-synthesis by protein polymer sequences. (a) UV-Vis spectrum of protein polymer-GNP nanocomposites at pH6 and pH8 (inset shows the templated-synthesis products of CE_1_-His_6_-GNP pH6 (I), E_1_C-His_6_-GNP pH6 (II), CE_1_-His_6_-GNP pH8 (III), E_1_C-His_6_-GNP pH8 (IV) and phosphate buffer-GNP pH8 (V)). TEM of (b) CE_1_-His_6_-GNP and (c) E_1_C-His_6_-GNP at pH8.

Both CE_1_-His_6_ and E_1_C-His_6_ were biosynthesized through recombinant bacterial expression and purified via nickel affinity resin. The protein diblock polymers were subject to GNP templated-synthesis without use of capping reagents. Gold salt (HAuCl_4_) solution was directly added to protein samples [24,25], followed by reduction with NaBH_4_ [43] under pH 6 and 8 (Figure 2a). Surprisingly, the P-GNP nanocomposites were stable at pH 8; within one week, absorbance spectra of complexes remained nearly the same with no observed precipitation even after one month when stored at room temperature (data not shown). The CE_1_-His_6_-GNP and E_1_C-His_6_-GNP exhibited successful templated-synthesis of GNPs with a distinct red-brown color change, confirmed by an observable peak at ~520 nm under pH 8 (Figure 2a). Since the lone pair electron on ε^2^N of histidine is protonated at pH ≤ 6, the protein polymer did not undergo GNP templated-synthesis very well under pH 6 conditions, consistent with literature [26]. Both CE_1_-His_6_ and E_1_C-His_6_ in the absence of gold salt did not lead to any detectable absorption peak at 520 nm (Figure S3); gold salt in the absence of protein did not produce signal indicating that the protein polymers were necessary for GNP templated-synthesis (Figure 2a). To affirm that the GNP templated-synthesis was due to the His_6_ tag, proteins lacking the N-terminal His_6_ sequence did not exhibit a strong signal at 520 nm (Figure S3).

### Morphological characterization of P-GNP nanocomposites

To assess the morphology and sizes of the P-GNP nanocomposites, transmission electron microscopy (TEM) was performed (Figures 2b and 2c). As expected [31], the CE_1_-His_6_-GNP and E_1_C-His_6_-GNP assembled into nanoparticles with diameters of 23.8 ± 5.6 nm and 23.9 ± 5.2 nm, respectively (Table 1 and Figure S4). Average diameters of GNPs in both CE_1_-His_6_-GNP and E_1_C-His_6_-GNP were 3.4 ± 0.9 nm and 3.5 ± 0.9 nm, respectively (Table 1 and Figure S5). Consistent with published work, the observed absorption peak at 520 nm is due to the GNP diameters being within 2–10 nm range (Figure S6) [44].

### Secondary structure analysis of P-GNP nanocomposites

**Figure 3:**
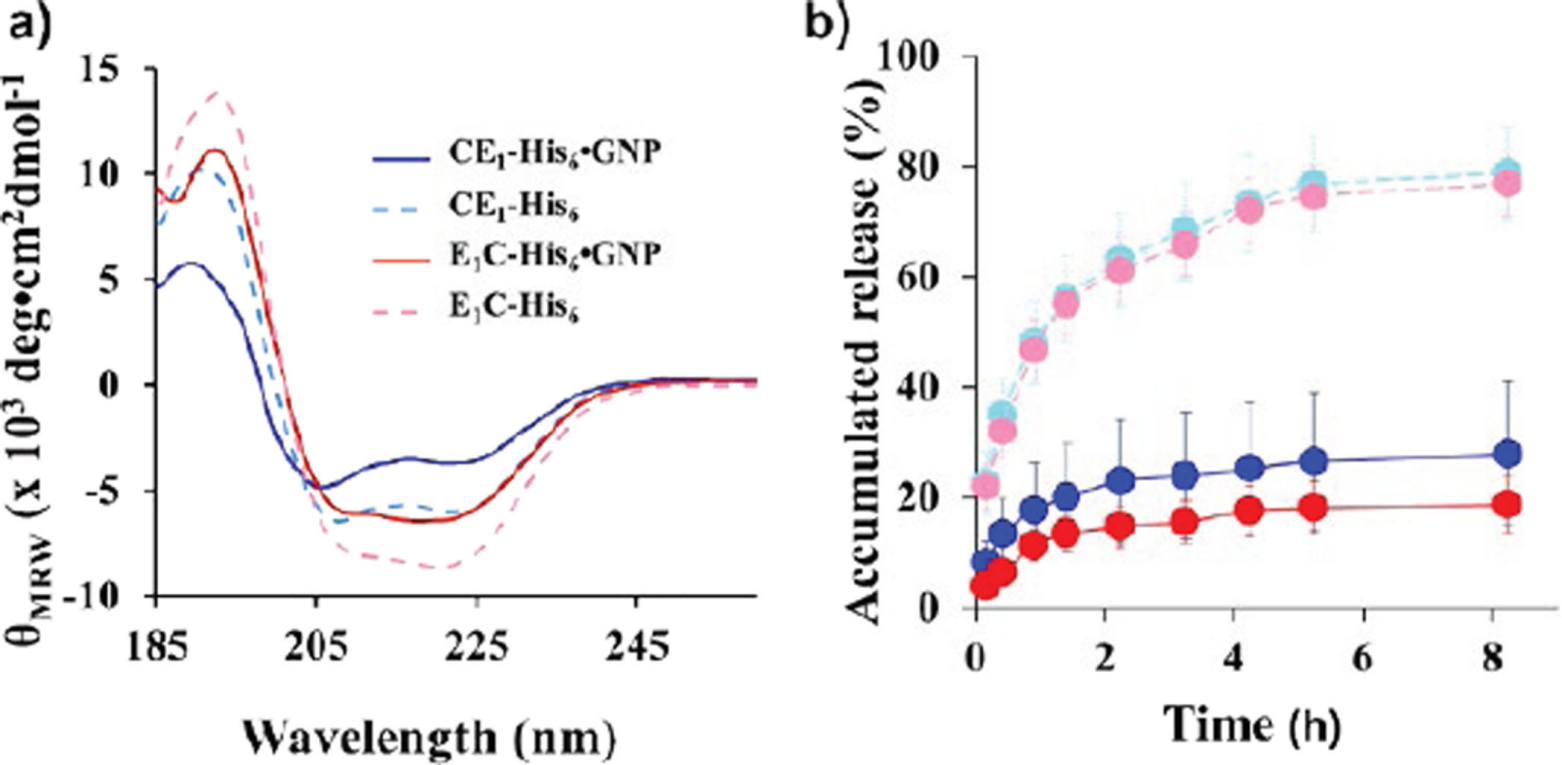
(a) CD wavelength scans of protein polymers in the absence and presence of GNP. (b) Accumulated release of CCM as a function of time.

A comparison of the secondary structures in the presence and absence of GNP was performed via circular dichroism (CD) to determine whether GNP templated-synthesis affected the protein polymer conformations (Figure 3a). While the overall shape of the wavelength scans were maintained, a slight loss in structure was observed for CE_1_-His_6_-GNP and E_1_C-His_6_-GNP relative to CE_1_-His_6_ and E_1_C-His_6_, respectively (Figures 3a, S7 and S8). To assess the impact of GNP templated-synthesis on the inverse temperature transition (T_t_), the UV/vis absorbance of CE_1_-His_6_-GNP and E_1_C-His_6_-GNP at 800 nm was monitored as a function of temperature (Table 1). Relative to the parent protein polymers, CE_1_-His_6_-GNP and E_1_C-His_6_-GNP revealed an increase in T_t_ by 11.2°C and 8.3°C, respectively.

### Curcumin loading and release

To evaluate the loading capacity of the protein polymers in the absence and presence of GNP, curcumin was incubated with CE_1_-His_6_, E_1_C-His_6_, CE_1_-His_6_-GNP and E_1_C-His_6_-GNP for 2 hours. Unbound curcumin was then separated and quantified to determine the amount of curcumin bound to the protein polymer and P-GNP complexes (Table 1). Surprisingly, CE_1_-His_6_-GNP exhibited higher binding capacity than CE_1_-His_6_ by 8 fold, while E_1_C-His_6_-GNP demonstrated a 7.3 fold improvement over E_1_C-His_6_.

Release studies were performed by loading the protein polymers and P-GNPs with curcumin and assessing the amount of free curcumin over time. The protein polymers alone released >50% curcumin after 1.4 hours; both CE_1_-His_6_ and E_1_C-His_6_ showed rapid and nearly complete release of 77.0% and 78.8% free curcumin by 8.25 hours (Figure 3b). By contrast, CE_1_-His_6_-GNP and E_1_C-His_6_-GNP, revealed a slow and sustained release of 27.9% and 18.8% free curcumin by 8.25 hours (Figure 3b). Thus, the P-GNP nanocomposites not only increased the binding capacity for curcumin but also, slowed down its release.

### Curcumin uptake by breast cancer cells

**Figure 4:**
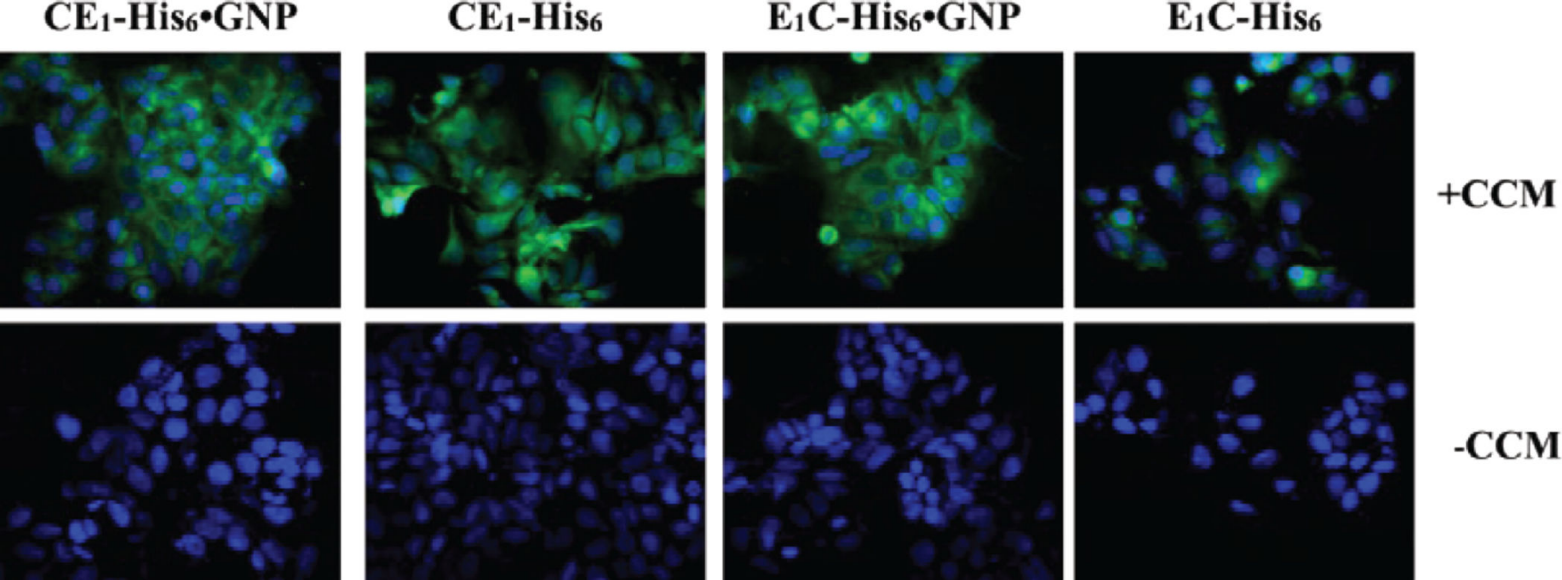
Fluorescence microscopy images of MCF-7 cells treated with protein polymers alone or P-GNP nanocomposites in the absence and presence of curcumin (CCM).

**Figure 5:**
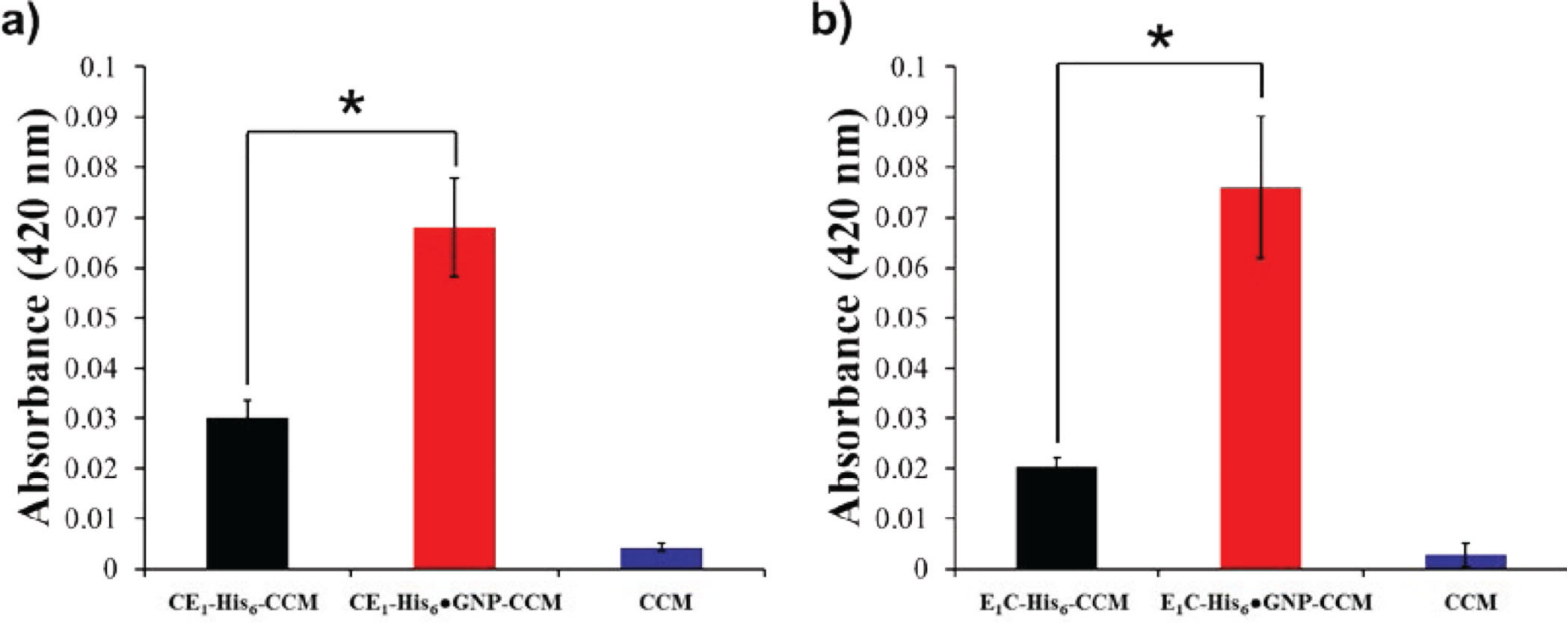
Quantification of uptake via extraction from cells. Absorption plots of (a) CE_1_-His_6_-CCM (black) CE_1_-His_6_-GNP-CCM (red), and CCM (blue) (p value < 0.05) and (b) E_1_C-His_6_ (black) E_1_C-His_6_-GNP-CCM (red) and CCM (blue) (p value < 0.05).

As curcumin is insoluble under aqueous conditions and does not effectively penetrate cancer cells alone [40], we investigated whether the P-GNP nanocomposites could enhance small molecule delivery and uptake by MCF7 breast cancer cells. Both CE_1_-His_6_-GNP and E_1_C-His_6_-GNP complexed with curcumin exhibited uptake as visualized by fluorescence (FITC channel); the curcumin appeared to be present in the cytoplasm as demonstrated by the overlay with DAPI stained cells (Figure 4). We also explored whether the protein polymers alone would deliver curcumin; both CE_1_-His_6_ and E_1_C-His_6_ revealed uptake albeit substantially less than the P-GNP nanocomposites (Figure 4). To assess whether CE_1_-His_6_-GNP, E_1_C-His_6_-GNP, CE_1_-His_6_ and E_1_C-His_6_ were themselves toxic to the cells, MTS assays were conducted; neither the protein polymer or P-GNP nanocomposites exhibited cytotoxicity (Table S3 and S4). Under identical conditions, the curcumin alone control did not show any uptake at the same concentrations of the protein polymers alone and the P-GNP nanocomposites. This was confirmed by quantifying curcumin extracted from the cells. Extraction of curcumin revealed 2.25-fold and 3.75-fold greater amount of available curcumin for CE_1_-His_6_-GNP and E_1_C-His_6_-GNP, respectively, relative to the protein polymers alone (Figure 5).

## Discussion

### Gold nanoparticle templated-synthesis influence on secondary structure and inverse temperature transition

We have produced stable P-GNP nanocomposites by GNP templated-synthesis through engineered N-terminal hexahistidine sequences within the protein diblocks CE_1_-His_6_ and E_1_C-His_6_. Either removing the hexahistidine sequence or decreasing the pH to alter the protonation state of the histidine residues does not lead to GNP production (Figure 2). After confirming GNP templated-synthesis to CE_1_-His_6_ and E_1_C-His_6_ spectroscopically, secondary structure analysis reveals that although a slight loss in alpha helicity is observed, the nanocomposites maintain overall conformation (Figure 3a). While *in situ* GNP templated-synthesis does not dramatically alter the protein polymer conformations, it does impact their thermoresponsive behavior. The marked effects on the thermoresponsiveness upon GNP templated-synthesis by the CE_1_-His_6_ and E_1_C-His_6_ protein polymers, regardless of the orientation of the domains can explain the improved loading capacity for curcumin. Upon GNP templated-synthesis, the P-GNP nanocomposites possessed elevated inverse temperature transitions (Table 1), indicative of heightened resistance to coacervative temperature-induced conformation changes. The enhanced stability against coacervation could impose greater mobility via increased hydration on the P-GNP nanocomposites thereby exposing more non-specific sites for curcumin binding leading to improved loading capacity.

### Small molecule binding properties after gold nanoparticle templated-synthesis and *in vitro* delivery

The C domain present in both diblocks CE_1_-His_6_ and E_1_C-His_6_ is capable of binding small hydrophobic molecules such as curcumin [31]. This phytochemical possesses medically relevant pharmacological properties yet it fails to remain stable under physiological conditions [29]. Therefore, maximizing curcumin loading capacities and optimizing slower release profiles in carriers would be important for drug delivery. Upon GNP templated-synthesis of both protein diblocks with gold nanoparticles, there is a 7.3 and 8-fold increase in curcumin binding for CE_1_-His_6_-GNP and E_1_C-His_6_-GNP, respectively, when compared to the protein polymers alone (Table 1). Curcumin is interesting in that it only exhibits fluorescence upon binding to other molecules [40]. The curcumin bound P-GNP nanocomposites show quenching and a blue shift in the fluorescence spectra suggesting a proximity effect of the GNP on the fluorescence properties of curcumin (Figure S9). This further affirms that the P-GNP nanocomposites are binding to the curcumin.

The P-GNP nanocomposites demonstrate a prolonged release profile whereby nearly 70% of available curcumin was retained within both the P-GNP nanocomposites after 8.25 hours (Figure 3b). In contrast, the protein polymers alone released more than 50% of retained curcumin after 1.4 hours. The prolonged release profile could be due to: i) the binding of curcumin to GNPs and ii) the stabilization or increase in T_t_ observed upon GNP templation as mentioned. Previous work has demonstrated the ability of GNPs to bind small molecules directly [45], suggesting that the enhanced binding capacity of the P-GNP nanocomposites for curcumin could be attributed to the GNPs. The improved binding and stability provided by the GNP templation (Table 1), could cause to the prolonged release profile. These results translate to successful delivery into MCF-7 cells.

While it is unclear whether CE_1_-His_6_, E_1_C-His_6_, CE_1_-His_6_-GNP and E_1_C-His_6_-GNP get taken up by the cells, there is an improved delivery of curcumin by the P-GNP nanocomposites. Extraction of curcumin from treated MCF-7 cells reveals a greater than 2-fold increase in bioavailable phytochemical by both the nanocomposites relative to their protein polymers counterparts (Figures 5a and 5b). The high amount of curcumin recovered from the cells implies chemical protection and half-life extension of the labile, yet biologically active curcumin.

## Conclusions

Remarkably, both CE_1_-His_6_-GNP and E_1_C-His_6_-GNP nanocomposites exhibit improved small molecule loading, slow and extended release as well as effective delivery when exposed to MCF-7 breast cancer cells. Further efforts are underway to elucidate the mechanisms by which P-GNP nanocomposites impact small molecule binding and releasing profile. These hybrid constructs can greatly broaden the biomaterials candidates for applications in targeted drug delivery. This can be achieved via the incorporation of tumor targeting domains in the solvent exposed residues of the protein polymer [46–48]. Furthermore, the drug loaded-nanocomposites, by way of templated-synthesis of GNP on the protein polymer, could be used for tandem chemotherapy and light-irradiated phototherapy [10,20,48].

## Supplementary Material

Supplementary file

## Figures and Tables

**Table 1:** Particle sizes, T_t_ and Loading capacities of proteins in the presence and absence of GNPs.

Composite	Size of protein particles (nm)	Size of GNPs (nm)	T_t_ (°C)	CCM/P^c^ molar binding ratio
CE_1_-His_6_	26.0 ± 3.0^a^	N/A	55.0 ± 0.8	0.40 ± 0.06
E_1_C-His_6_	27.9 ± 3.7^a^	N/A	33.8 ± 2.2	0.41 ± 0.10
CE_1_-His_6_- GNP	23.8 ± 5.6^b^	3.4 ± 0.9	66.2 ± 0.8	3.16 ± 0.44
E_1_C-His_6_- GNP	23.9 ± 5.2^b^	3.5 ± 0.9	42.1 ± 7.1	2.95 ± 0.42

a Data from (ref 28).

b Sizes were measured on P-GNP nanocomposites from > 130 particles.

c Ratio of Curcumin to protein or P-GNP.
