# Supplementary material for: Engineered Protein Polymer-Gold Nanoparticle Hybrid Materials for Small Molecule Delivery
Source: J Nanomed Nanotechnol. Author manuscript; Available in PMC 2016 Apr 12. (PMC4828936; doi:10.4172/2157-7439.1000356)
Supplement: Supplementary file [file NIHMS769407-supplement-Supplementary_file.pdf]

Supporting Information

Engineered Protein Polymer-Gold Nanoparticle Hybrid Materials for Small Molecule Delivery

Min Dai[1#], Frezzo JA[1#], Sharma E[1], Chen R[1], Singh N[1], Yuvienco C[1], Caglar E[2], Xiao S[2], Saxena A[2], Montclare JK[1,3,4*]

[1]*Department of Chemical and Biomolecular Engineering, NYU Tandon School of Engineering, Brooklyn, New York 11201, USA*

[2]*Department of Biology, Brooklyn College and Graduate Center, City University of New York, Brooklyn, New York 11210, USA*

[3]*Department of Chemistry, New York University, New York, New York 10003, USA*

[4]*Department of Biochemistry, SUNY Downstate Medical Center, Brooklyn, New York 11203, USA*

[#]*These authors contributed equally*

***Corresponding author:** Montclare JK, Department of Chemical and Biomolecular Engineering, NYU Tandon School of Engineering, Brooklyn, New York 11201, USA, Tel: 1-646-997-3679; E-mail: montclare@nyu.edu

CE_1-His_6	MRGSHHHHHHGSACELAATATATATATATAACGDLAPQMLRELQETNAALQDVRLLRQQVKEITFLKNTVMESDASGLQLLRQQVKEITFLKNTVMES
CE_1-IEGR	MRGSHHHHHHIEGRELAATATATATATATAACGDLAPQMLRELQETNAALQDVRLLRQQVKEITFLKNTVMESDASGLQLLRQQVKEITFLKNTVMES
E_1C-His_6	MRGSHHHHHHGSKPIAASAVPGVGVPGVGVPGFGVPGVGVPGFGVPGVEVPGVEVPLEGSELAATATATATATATAACGDLAPQMLRELQETNAAL
E_1C-IEGR	MRGSHHHHHHIEGRIAASAVPGVGVPGVGVPGFGVPGVGVPGFGVPGVEVPGVEVPLEGSELAATATATATATATAACGDLAPQMLRELQETNAAL

CE_1-His_6	DASGLQQATATATATATATAVDKPIAASAVPGVGVPGVGVPGFGVPGVGVPGFGVPGVEVPGVEVPLEGSGTGAKL
CE_1-IEGR	DASGLQQATATATATATATAVDKPIAASAVPGVGVPGVGVPGFGVPGVGVPGFGVPGVEVPGVEVPLEGSGTGAKL
E_1C-His_6	QDVRLLRQQVKEITFLKNTVMESDASGLQAATATATATATATAVDLQPS
E_1C-IEGR	QDVRLLRQQVKEITFLKNTVMESDASGLQAATATATATATATAVDLQPS

Figure S1. Alignment of protein sequences translated from DNAs that were verified by DNA sequencing at Eurofins. His tag cleavage site IEGR is highlighted with underline in CE_1-IEGR and E_1C-IEGR.

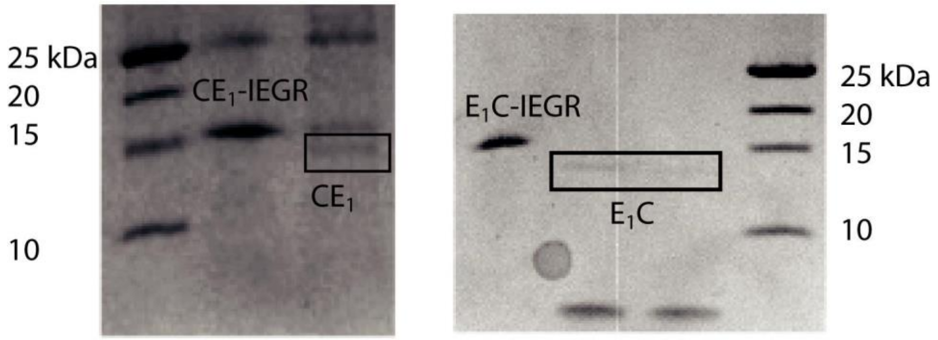

Figure S2: 12% SDS-PAGE verified cleavage of CE_1 -IEGR and E_1 C-IEGR on IEGR site by Factor Xa. Molecular weight of CE_1-IEGR and E_1C-IEGR are 14150.95 and 13950.75 Da respectively. After His tag and IEGR site removal, molecular weight of CE_1 and E_1C are 12441.08 and 12240.88 Da respectively.

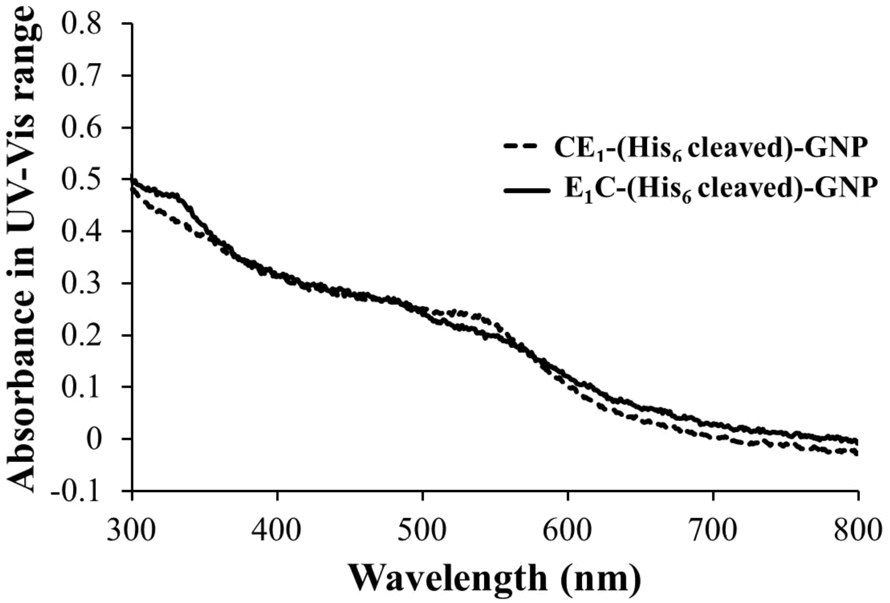

Figure S3: Wavelength spectra of CE_1-His_6-GNP (blue), E_1C-His_6-GNP (red), CE_1-(His_6 cleaved)-GNP (green) and E_1C-(His $_6$ cleaved)-GNP (purple). The spectra for phosphate buffer supplemented with Factor XA cleavage buffer and GNP was subtracted from each spectra.

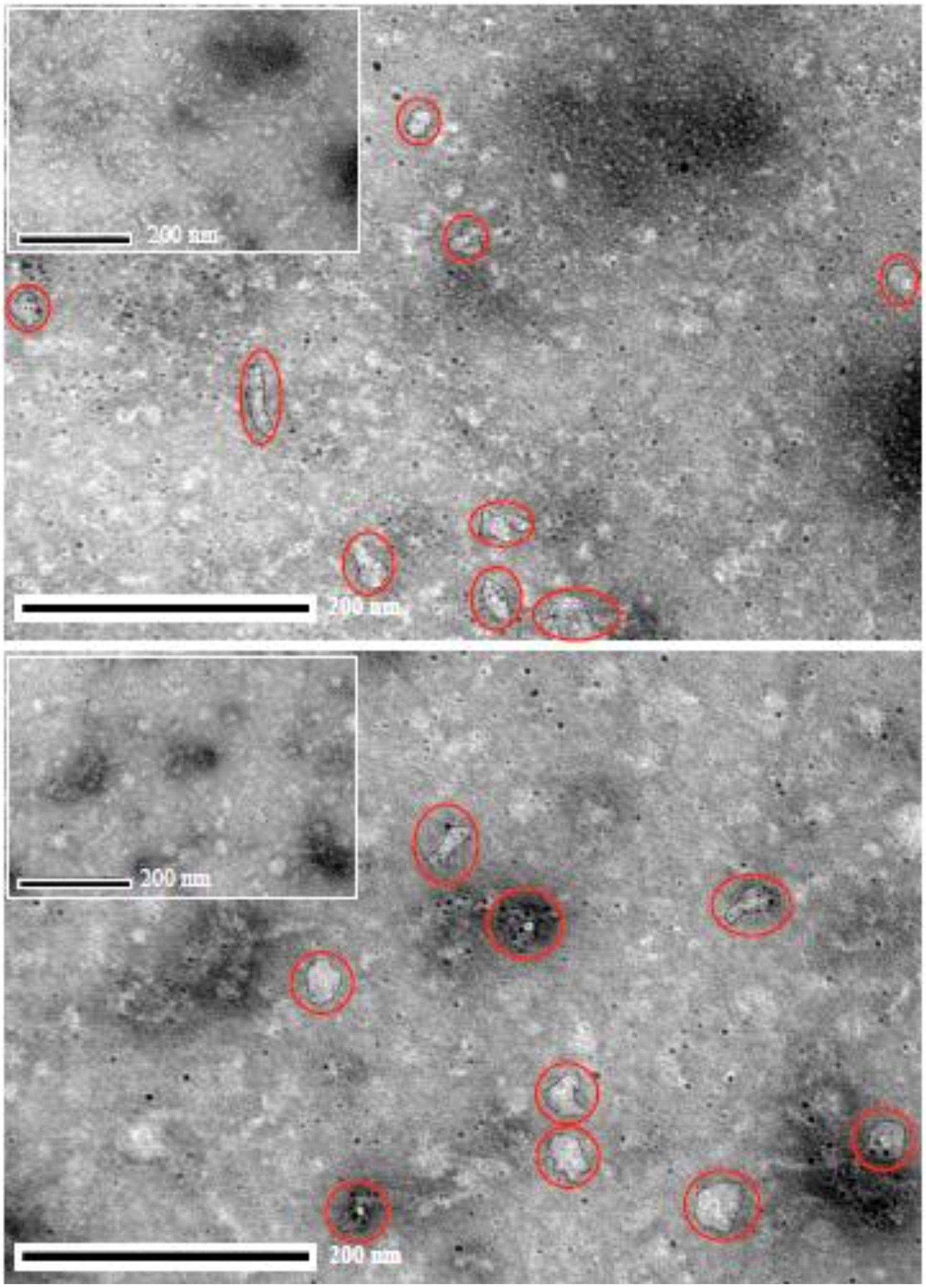

Figure S4: Protein size measurement. Micrographs of CE_1-His_6-GNP (top) and E_1C-His_6-GNP (bottom) with protein particles highlighted (with black circle). Selected protein particles are analyzed for size measurements using Image.

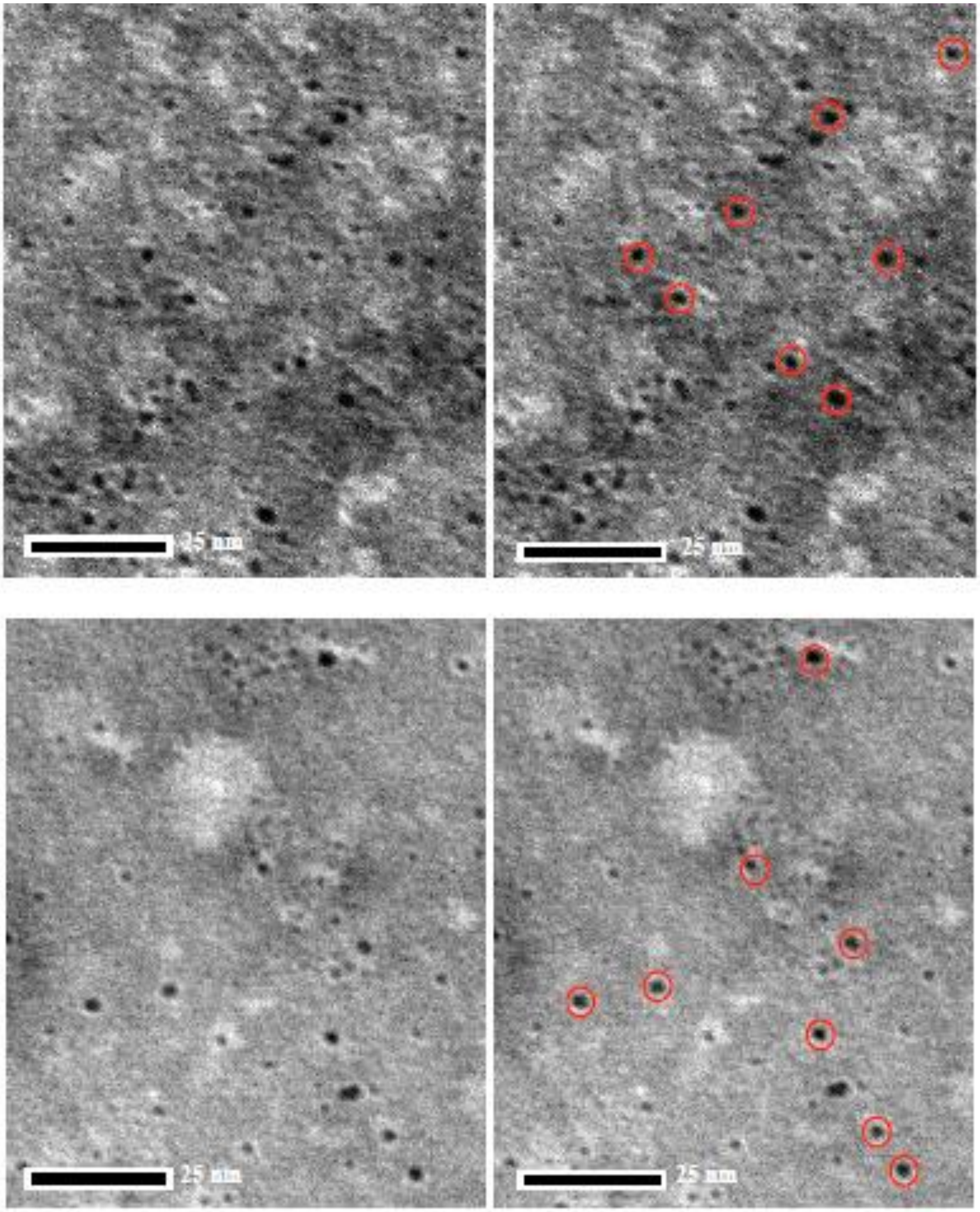

Figure S5: Micrographs of CE_1-His_6-GNP (upper panel) and E_1C-His_6-GNP (lower panel) samples with selected GNPs (with red circle, right side)) for size measurements using Image.

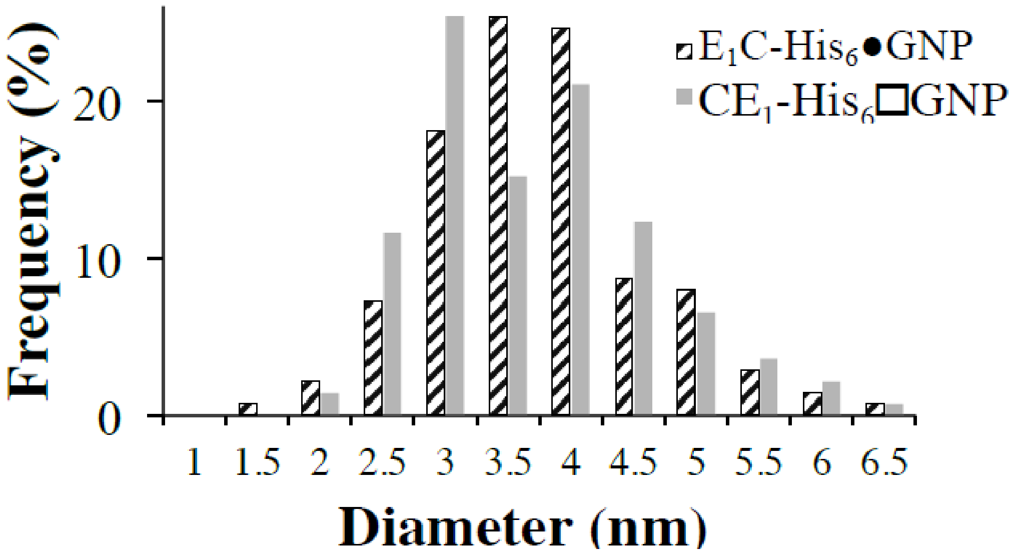

Figure S6: Size distribution of GNPs in each protein constructs. More than 130 particles were analyzed for both constructs. The average diameter of GNPs in $E_1C\text{-}His_6$-GNP is 3.5 ± 0.9 nm and 3.4 ± 0.9 nm in $CE_1\text{-}His_6$-GNP.

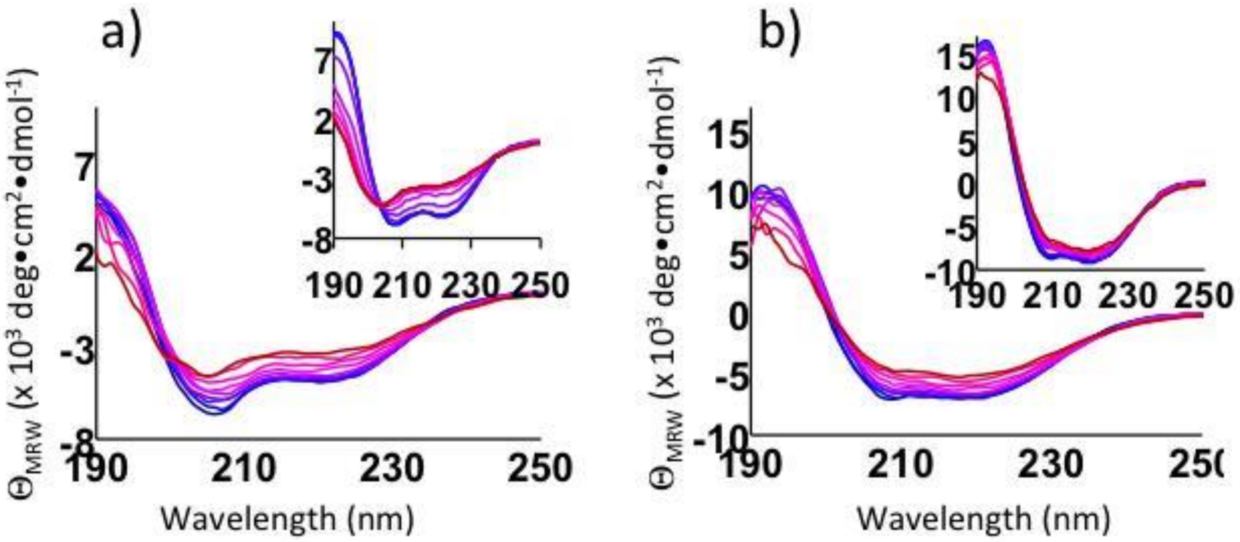

Figure S7: Temperature dependent wavelength scans of (a) $CE_1\text{-}His_6$-GNP and (b) $E_1C\text{-}His_6$-GNP from 20ºC to 95ºC. Insets represent the temperature-dependent wavelength scans of the same protein in the absence of GNP templated-synthesis.

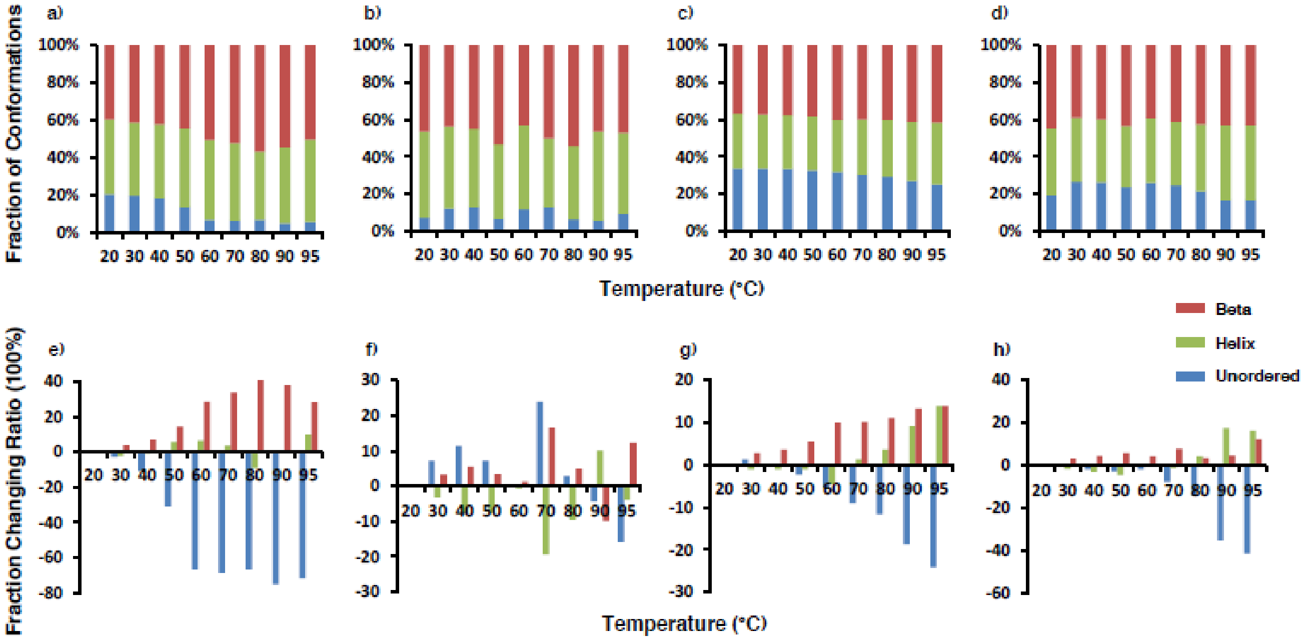

Figure S8: Secondary structure calculation using CDSSTR of (a) CE_1 -His_6, (b) CE_1-His_6-GNP, (c) E_1C-His_6 and (d) E_1C-His_6-GNP at pH 8.0. The fraction of secondary structure ratios as a function of temperature for (e) CE_1-His_6, (f) CE_1 -His_6 -GNP, (g) E_1C- His_6 and (h) E_1 C-His_6-GNP via CDSSTR.

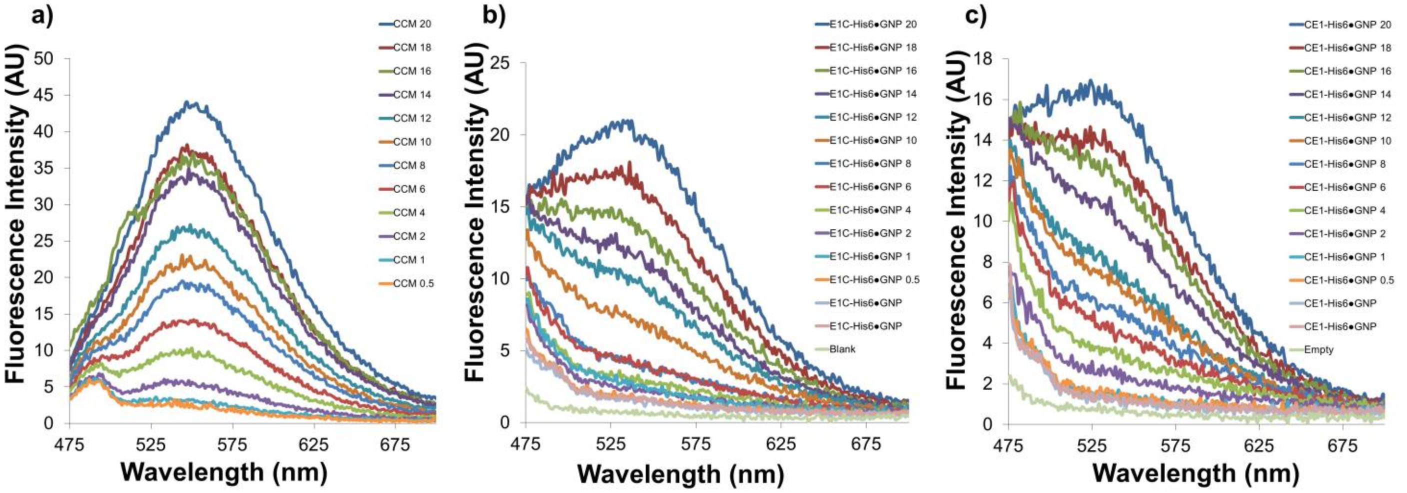

Figure S9: Fluorescence, Ex: 420 nm; optical cutoff: 455 nm, of (a) CCM, (b) E_1C-His_6-GNP and (c) CE_1-His_6-GNP. Values following each data point represent the micromolar concentration of CCM.

	CE_1-His_6	CE_1-His_6•GNP	E_1C-His_6	E_1C-His_6•GNP
Concentration of protein	0.1621 mg/mL	0.1621 mg/mL	0.1818 mg/mL	0.1818 mg/mL
Concentration of NaCl	0.946 M	0.946 M	0.45 M	0.45 M

Table S1. Final concentration of protein and NaCl in samples for T_t measurement

	CE_1-His_6	CE_1-His_6•GNP	CE_1-His_6-CCM1[a]	CE_1-His_6-GNP-CCM2[a]	CCM2[b]
Conc. of protein	10	10	10	10	N/A
Conc. of CCM	N/A	N/A	4.27	46.32	46.32
	E_1C-His_6	E_1C-His_6-GNP	E_1C-His_6-CCM3[a]	E_1C-His_6-GNP-CCM4[a]	CCM4[b]
Conc. of protein	10	10	10	10	N/A
Conc. of CCM	N/A	N/A	1.58	40.1	40.1

[a]CCM added in uptake experiment are equivalent to the loading capacities of each protein sample

[b]For CCM controls, the amount equivalent to P-GNP loading capacities was used

Table S2: Final concentration (μM) of each component in samples for cell uptake experiment.

	CE_1-His_6	CE_1-His_6-GNP	CE_1-His_6-CCM1	CE_1-His_6-GNP -CCM2	CCM1	CCM2	Cell only
Abs.	2.07 ± 0.01	2.028 ± 0.01	2.046 ± 0.01	2.046 ± 0.01	1.999 ± 0.05	2.025 ± 0.02	2.045 ± 0.01
	E_1C-His_6	E_1C-His_6-GNP	E_1C-His_6-CCM1	E_1C-His_6-GNP -CCM2	CCM1	CCM2	DMEM
Abs.	2.053 ± 0.00	2.042 ± 0.02	2.034 ± 0.02	2.042 ± 0.01	2.039 ± 0.00	2.044 ± 0.01	0.00 ± 0.00

Table S3: MTS Assay after 4 Hour Treatment.

	CE_1-His_6	CE_1-His_6-GNP	CE_1-His_6-CCM1	CE_1-His_6-GNP -CCM2	CCM1	CCM2	Cell only
Abs.	2.138 ± 0.01	2.051 ± 0.01	2.041 ± 0.00	2.026 ± 0.03	2.034 ± 0.02	1.999 ± 0.01	2.017 ± 0.05
	E_1C-His_6	E_1C-His_6-GNP	E_1C-His_6-CCM1	E_1C-His-GNP -CCM2	CCM1	CCM2	DMEM
Abs.	2.119 ± 0.00	1.999 ± 0.02	2.034 ± 0.00	1.866 ± 0.15	1.974 ± 0.01	1.98 ± 0.04	0.00 ± 0.00

Table S4: MTS Assay after 24 Hour Treatment.
